# Respiratory Involvement in Patients with Neuromuscular Diseases: A Narrative Review

**DOI:** 10.1155/2019/2734054

**Published:** 2019-12-26

**Authors:** Athanasios Voulgaris, Maria Antoniadou, Michalis Agrafiotis, Paschalis Steiropoulos

**Affiliations:** ^1^MSc Program in Sleep Medicine, Medical School, Democritus University of Thrace, Alexandroupolis, Greece; ^2^Department of Pneumonology, Medical School, Democritus University of Thrace, Alexandroupolis, Greece; ^3^Department of Pneumonology, General Hospital “G. Papanikolaou”, Thessaloniki, Greece

## Abstract

Respiratory muscle weakness is a major cause of morbidity and mortality in patients with neuromuscular diseases (NMDs). Respiratory involvement in NMDs can manifest broadly, ranging from milder insufficiency that may affect only sleep initially to severe insufficiency that can be life threatening. Patients with neuromuscular diseases exhibit very often sleep-disordered breathing, which is frequently overlooked until symptoms become more severe leading to irreversible respiratory failure necessitating noninvasive ventilation (NIV) or even tracheostomy. Close monitoring of respiratory function and sleep evaluation is currently the standard of care. Early recognition of sleep disturbances and initiation of NIV can improve the quality of life and prolong survival. This review discusses the respiratory impairment during sleep in patients with NMDs, the diagnostic tools available for early recognition of sleep-disordered breathing and the therapeutic options available for overall respiratory management of patients with NMDs.

## 1. Introduction

Neuromuscular diseases (NMDs) encompass a diverse group of diseases which may affect the upper motor neurons, lower motor neurons, peripheral nerves, the neuromuscular junction, and muscles. Although this definition may include a large variety of disorders, the term “neuromuscular” is reserved for inherited or acquired diseases predominately manifesting with motor dysfunction. From the pulmonologist's perspective all NMDs present with a common trait: impairment of ventilatory function. This impairment is caused by either compromised airway patency and protective reflexes and/or by reduced respiratory pump efficiency. These pathophysiological events result in sleep-disordered breathing, followed by diurnal hypoventilation at later disease stages. In addition, patients with NMDs are prone to developing various other respiratory complications such as infections, atelectasis, and aspiration syndromes [1, 2]. The introduction of ventilatory support in the early stages of symptomatic sleep hypoventilation, especially in the form of noninvasive ventilation (NIV), as well as various other advances in respiratory care have contributed significantly to the improvement of survival and the quality of life of patients with NMDs [3–6].

NMDs can be hereditary or acquired, slowly or rapidly progressive [7, 8]. Inherited NMDs are not uncommon, with an estimated prevalence exceeding 1 in 3000 among newborns [9]. The prevalence of the majority of NMDs generally ranges between 1 and 10/100000 population; however some disorders are much more common e.g. postpolio syndrome and Charcot-Marie-Tooth disease with a prevalence greater than 10/100000, whereas others are quite rare, e.g. Lambert-Eaton myasthenic syndrome and congenital muscular dystrophies with a prevalence of less than 1/100000 [10]. In a European survey, among patients treated with domiciliary mechanical ventilation, neuromuscular patients were most likely to receive prolonged (>6 years) ventilatory support and to undergo tracheostomy procedures [11].

NMDs cause respiratory disease mainly by causing respiratory muscle dysfunction. Respiratory insufficiency had been the main cause of morbidity and mortality in patients with NMDs until recent respiratory care became available [12, 13]. Respiratory complications and failure will eventually develop in the majority of the patients with NMDs although the age of onset might vary depending on the rate of disease progression. For example, most of the children with spinal muscular atrophy (SMA) type 1 will develop respiratory failure and die before the age of 3 years without adequate respiratory management, while respiratory failure will present later in childhood in only 40% of patients with SMA type 2 [14, 15]. In patients with Duchenne muscular dystrophy (DMD), respiratory failure commonly appears after the loss of ambulation [16]. Conversely, respiratory failure can precede non respiratory muscle weakness, in patients with Pompe's disease and amyotrophic lateral sclerosis [17, 18].

NMDs can also present with various non respiratory manifestations. DMD may cause left ventricular insufficiency and conduction abnormalities [19]. Patients with NMDs may also manifest bony deformities such as chest wall and spine abnormalities. In DMD, scoliosis commonly presents during the second decade of life, after patients become wheelchair-bound, as a result of paravertebral muscle atonia [20]. Conversely, and unless ventilatory support is provided, patients with SMA types 1 and 2 develop bell-shaped chest deformity and pectus excavatum attributed to the preferential involvement of intercostal muscles and the relative sparing of the diaphragm [21]. Patients with myotonic dystrophy commonly exhibit a mild cognitive impairment and a narcolepsy-like clinical phenotype, associated with excessive daytime sleepiness without adequate management for SDB [22, 23].

## 2. Respiratory Function in Patients with Neuromuscular Diseases

From a practical point of view, the respiratory muscles can be grouped into inspiratory (main and accessory), expiratory, and the upper airway muscles. Dysfunction of these groups can manifest as three different types of failure: (1) respiratory pump failure (inspiratory muscles' dysfunction) (2) upper airway failure, and swallowing dysfunction (upper airway muscles' dysfunction) and (3) cough failure (inspiratory, expiratory, and upper airway muscles dysfunction) [1, 13].

The respiratory pump is responsible for moving the air in and out of the lungs. The respiratory pump fails when it operates against an increased workload or when the respiratory muscles fail to generate enough force. Both of these circumstances predispose to respiratory muscle fatigue, which is defined as the inability to sustain a contractile force against a constant workload [24]. As fatigue increases the patient develops a rapid shallow breathing pattern, which is not fatigue-specific, but rather a reflex response to increased work of breathing [25].

In patients with NMDs the contractile function of the inspiratory and expiratory muscles is impaired. Moreover, chest wall compliance is decreased as a result of scoliosis and stiffening of the thoracic cage tendons and ligaments, while recurrent aspirations, microatelectasis, and pulmonary congestion due to cardiac disease are responsible for a decrease in lung compliance [26]. In addition, the upper airway collapsibility and hypotonia lead to increased resistance to airflow, which also contributes to the increased workload of the failing respiratory pump [27]. Although the initial physiologic response to these events is an increase in respiratory drive, this mechanism is eventually insufficient to maintain alveolar ventilation and hypoventilation finally ensues [28, 29]. Moreover, upper airway muscle failure also accounts for swallowing dysfunction and impaired airway protection, leading to recurrent episodes of aspiration and pneumonia [13].

Normal cough consists of an initial inspiratory phase to reach a lung volume equal to 85–90% of TLC; a compression phase characterized by rapid closure of the glottis for 0.2 seconds with simultaneous contraction of the expiratory muscles to create a pressure greater than 200 cm H_2_O; and an expulsion phase characterized by rapid opening of the glottis while the expiratory muscles continue to contract in order to produce an expiratory cough flow greater than 400 L/min [30]. High cough flows are essential for removing secretions and debris and clearing the airways. Therefore, impairment of either the inspiratory, expiratory or glottic muscles in patients with NMDs might compromise effective cough function leading to increased susceptibility to respiratory infections, atelectasis and respiratory failure [1, 31].

## 3. Respiratory Function during Sleep in Patients with Neuromuscular Diseases

Normal sleep is characterized by a decrease in alveolar ventilation by 1-2  L/min mainly caused by a decrease in tidal volume, while the pattern of breathing becomes shallow and irregular [32]. As a result PCO_2_ increases and O_2_ decreases by 2–8 mmHg each, while the ventilatory response to hypoxemia and hypercapnia is attenuated [33, 34]. These changes become more pronounced during deep sleep and especially during phasic REM sleep [32]. There are several reasons accounting for these physiologic events, including the reduced lung volume in the supine position, the withdrawal of the wakefulness drive to breathe and the reduced chemosensitivity of respiratory control center [35]. In addition, the tone of the intercostal muscles decreases during sleep [36], while the tone of the diaphragm decreases during NREM but is maintained during REM sleep; as a result, normal ventilation during REM sleep becomes totally dependent on the diaphragmatic function [37]. Finally, the activity of the pharyngeal dilator muscles -particularly of the genioglossus- decreases gradually during sleep resulting in increased upper airway resistance which becomes maximal during REM sleep [38].

These events are more pronounced in patients with NMDs as a result of respiratory and upper airway muscle weakness. The most frequent breathing disorder in NMDs is hypoventilation presenting at first during REM sleep and then occurring at all sleep stages and finally when the patient is awake [39, 40]. Symptoms suggestive of nocturnal hypoventilation are sudden arousals, morning headache, daytime sleepiness, and fatigue [41].

The most common respiratory event during sleep in NMDs is central hypopnea [42–45]. Central hypopneas are most commonly observed during phasic REM sleep, as a result of the decreased tone of the intercostal and accessory muscles combined with inadequate function of the diaphragm [42, 44–46]. Obstructive events are also observed in patients with NMDs, especially in DMD and acid maltase deficiency. In these disorders, sleep disordered breathing follows a bimodal distribution with obstructive events occurring early in the course of the disease while frank hypoventilation prevails at later years [43, 47]. In addition, obstructive events do not appear to be more likely in bulbar ALS patients, although the evidence here is equivocal [48, 49]. Some studies suggest that obstructive events are more likely in patients with a higher body mass index (BMI), in snorers, in those with anatomic abnormalities such as macroglossia, and in those with increased upper airway resistance during REM sleep [42, 43, 50]. Furthermore, Cheyne-Stokes breathing and other central sleep apnea syndromes in association with cardiomyopathy and unstable control of breathing have also been reported [51, 52]. During sleep, patients with NMDs also exhibit hypoxemia which is related to the presence of hypoventilation, apneas and hypopneas, and ventilation-perfusion mismatch due to atelectasis occurring in the supine position [53].

## 4. Evaluation of Respiratory Function and Cough in Patients with Neuromuscular Diseases

Respiratory assessment in patients with NMDs includes evaluation of respiratory muscle strength, gas exchange, and cough efficiency. Spirometry, arterial blood gas, mouth and nasal pressures are among the most widely used techniques.

In NMDs impairment of respiratory muscle contractile function leads to respiratory pump failure, characterized by decreases in vital capacity (VC), total lung capacity (TLC) and functional residual capacity (FRC) [54]. This reduction in VC is further augmented in the supine position and a drop of greater than 25% is considered 90% sensitive and 79% specific for the diagnosis of diaphragmatic weakness [55]. Interestingly, a decrease in VC can be observed earlier in the course of the disease as compared to TLC, due to the increased residual volume (RV) observed in patients with expiratory muscle weakness [56]. However, although VC is considered a robust index for the respiratory evaluation of patients with NMDs, it can be fairly insensitive in early disease stages. In a study involving patients with various types of NMDs, an inspiratory VC of less than 60% predicted the presence of sleep apnea syndrome and a VC of less than 40% was associated with sleep hypoventilation with a sensitivity of 91% and 94% and a specificity of 89% and 79% respectively; patients with a VC of less than 25% suffered from daytime ventilatory failure [39]. In a cohort involving more than 1000 patients with ALS, forced vital capacity (FVC) less than 75% was associated with a median survival of 2.91 years as opposed to 4.08 years for patients with FVC of greater than 75% [57]. In another study, a supine FVC of less than 80% was 94.6% sensitive in predicting death or tracheostomy within the first year in a group of patients with ALS [58]. In patients with DMD, VC increases until the age of 10–12 years, when it reaches a plateau, followed by a decline which can be as high as 10.7% per year [59]. Loss of ambulation and establishment of scoliosis is associated with an accelerated decline in lung function in DMD patients and a VC of less than 1L predicts a 5-year survival rate of 8% without adequate noninvasive respiratory support [8, 60, 61]. Moreover, an inspiratory VC of less than 1.1 L could reliably identify patients at high risk for severe chest infections in a retrospective study involving children and adolescents with various NMDs [62].

An increased PCO_2_ value in arterial blood gas indicates the presence of diurnal respiratory pump failure. In addition, an increased alveolar-arterial difference should point towards a concurrent parenchymal lung disease. Of note, PCO_2_ commonly appears reduced at initial disease stages but increases later when respiratory muscle impairment has progressed significantly [63]. Importantly, either diurnal hypercapnia or an increased bicarbonate or base excess (BE) level might indicate coexistent nocturnal hypoventilation. In a study of patients with DMD, values of PCO_2_  > 45 mmHg and BE > 4 mmol/L predicted nocturnal hypoventilation with a sensitivity of 99% and 55% and a specificity of 75% and 100% respectively [64].

Cough efficiency is assessed by measuring cough flow variables, of which the most important are peak expiratory cough flow (PECF) and cough volume acceleration (CVA). Either a customary handheld peak flow meter, which is used for patients with asthma, or an office spirometer, can be used for cough flow measurements; however it should be noted that significant differences between pneumotachograph- and peak flow meter-based assessments have been reported [65]. Nevertheless, an inefficient cough is characterized by a lower peak inspiratory cough flow, a longer compression phase and a lower PECF [66].

PECF is the highest cough flow obtained after instructing the patient to take a deep breath and cough forcibly. For adults and older children, a PECF of less than 160 L/min is associated with ineffective secretion clearance and tracheostomy decannulation failure [67]. Moreover, a PECF less than 270 L/min is associated with higher risk for acute respiratory failure and higher respiratory morbidity during the course of a trivial respiratory tract infection [68–70]. Most authorities would consider a PECF less than 270 L/min as an indication for commencement of cough augmentation strategies [71, 72].

CVA is derived by dividing PECF by the time required to reach its value (peak expiratory flow rise time, PEFRT). In a prospective study involving stable ALS patients, a CVA of less than 28.9 L/sec^2^ was slightly more reliable than PECF in predicting ineffective cough during a respiratory tract infection [69]. In addition, when compared to PECF and PEFRT, CVA was the most reliable predictor of aspiration in patients with ALS [66]. In another study, the strength of cough in patients with ALS correlated with the ability to produce transient cough spikes during a maximal flow-volume maneuver; the absence of these spikes also correlated with increased mortality [73, 74].

Maximum insufflation capacity (MIC) and lung insufflation capacity are also important variables in the assessment of cough function. MIC is defined as the maximum volume of air that can be held in the lungs while the glottis is closed and is obtained by instructing the patient to stack several volumes of air after taking a deep breath. Air stacking can be performed with the use of a volume ventilator or a resuscitation bag with an one way valve, or with the technique of glossopharyngeal breathing [75]. When the glottic function is not intact, the same effect can be obtained via the use of a manual resuscitator with the expiratory port blocked, or a volume ventilator set to deliver volumes close to the subject's inspiratory capacity or a mechanical insufflator-exsufflator device (MI-E); in that instance the term lung insufflation capacity is preferred (LIC) [76]. MIC/LIC can be measured with a simple spirometer and the greater the difference between VC and MIC/LIC, the higher the PECF that will be achieved when the patient coughs from maximum insufflation [72, 76, 77].

Measurements of maximal static mouth pressures are among the most common methods used to assess inspiratory and expiratory muscle strength in daily clinical practice. They are measured via a mouthpiece, which is connected to a manometer. One limitation of the static mouth pressures is that they cannot be measured reliably in patients with facial muscle weakness [78]. Maximum inspiratory pressure (MIP) is the average pressure sustained over 1 sec when the patient takes a deep breath against a blocked mouthpiece from the RV (Mueller maneuver). MIP correlates well with VC in children and adults with various types of NMDs [39, 79–81]. When evaluated in a mixed population of children and adolescents with NMDs, a MIP less than −46 cm H_2_O predicted the presence of sleep apnea and a MIP less than −40 cm H_2_O was associated with sleep hypoventilation with a sensitivity of 82 and 95% and specificity of 89% and 65%, respectively [39]. Unlike VC, MIP is neither related to the number of chest infections nor the total days of antibiotic therapy during the preceding year [62].

Maximal expiratory pressure (MEP) is the average pressure sustained over 1 sec when the subject blows against a blocked mouthpiece from TLC (Valsava maneuver). A MEP greater than +60 H_2_O is required to produce an efficient cough, while a MEP of less than +45 H_2_O suggests impaired expiratory muscle function and decreased ability to cough [82]. In a study involving children and adolescents with DMD, MEP correlated with VC, but its rate of decline during the course of the disease was higher as compared to MIP and VC [81]. MEP has also been shown to correlate with the number of infections and the number of days on antibiotic therapy in the preceding year, although the correlation is weaker than for VC and PECF [62].

Nasal pressures are measured via a plug wedged into the nostril and connected to a manometer, while the subject performs a quick sniff, i.e., a sharp voluntary inspiratory maneuver through the nostril (sniff nasal inspiratory pressure, SNIP). This maneuver is performed from FRC and although it might be easier to perform and more suitable for patients with facial muscle weakness, more than 10 attempts are often required before a plateau is reached [78, 83]. SNIP has a strong correlation with PCO_2_ in nonbulbar patients with ALS and a SNIP of less than −40 cm H_2_O was the most reliable noninvasive predictor of hypercapnic respiratory failure in nonbulbar ALS patients with a sensitivity of 85% and a specificity of 81% [63, 84]. In a prospective study of 98 patients with ALS, a SNIP < −40 cm H_2_O was significantly correlated with the severity of nocturnal hypoxemia, while no such association was established for MIP and VC; in the same study, a SNIP < −40 cm H_2_O was associated with a median survival of 6 months [85]. In a more recent study of patients with ALS, a SNIP of less than −34 cm H_2_O could predict death or tracheostomy within one year with a sensitivity of 75% and a specificity of 72% [86]. Another study evaluated the evolution of MIP, SNIP, and VC in patients with DMD and Becker muscular dystrophy (BMD), between 5 and 20 years old. In patients with DMD, MIP remained stable, while VC and SNIP decreased consistently after an initial increase; however, SNIP showed an earlier decline than VC (at 10.5 years versus 12.5 years, respectively) [87].

## 5. Evaluation of Sleep-Disordered Breathing in Patients with Neuromuscular Diseases

Polysomnography (PSG) complemented by capnography is the gold standard method for the assessment of sleepdisordered breathing in patients with neuromuscular disorders, and overnight PSG should be applied when available [14, 16, 20, 88]. The American Thoracic Society recommends annual evaluation of sleep disordered breathing in children with DMD, ideally using PSG with simultaneous measurement of CO_2_ [16, 20]. However, in younger children with uncertain course of the disease or with rapid clinical deterioration or in patients with recurrent infections, sleep monitoring should be performed more frequently [16, 20, 88]. Nevertheless, and especially in cases where polysomnography is not available, other types of sleep studies such as cardiorespiratory polygraphy or pulse oximetry should be considered although these may not be sensitive enough to detect sleep-disordered breathing in NMDs [88, 89]. Indeed, although a “sagging” oximetry profile is a feature of sleep hypoventilation in patients with NMDs, short or mild episodes or hypoventilation might be missed by overnight pulse oximetry; however, some authorities assert that clinical significant hypoventilation is unlikely in asymptomatic patients who do not desaturate below 93% [88, 90]. Nevertheless, according to several authors, a desaturation to a value <88% (or 90%) for 5 consecutive minutes or a desaturation to a value <90% for 10% (or 30%) of the total recording time might be indicators of sleep hypoventilation in neuromuscular patients [90–93]. The addition of capnography might increase the diagnostic sensitivity of oximetry and the use of oxicapnometry could be considered as a more practical approach for neuromuscular patients when polysomnography is not available [88, 94–96]. According to the American Academy of Sleep Medicine scoring rules, sleep hypoventilation in adults is defined as 1) an increase in PCO_2_ (arterial, transcutaneous or end-tidal) to a value >55 mmHg for ≥10 minutes or (2) an increase in PCO_2_ by >10 mmHg, to a value >50 mmHg for ≥10 minutes. In children, sleep hypoventilation is scored when PCO_2_ exceeds 50 mmHg for >25% of total sleep time [97]. Some authors have employed alternative definitions of sleep hypoventilation, including a peak transcutaneous PCO_2 _> 49 mmHg [93] or a mean transcutaneous PCO_2_ > 50 mmHg [3, 15]. However, because various definitions are used for sleep hypoventilation [98], levels of CO_2_ approaching 50 mmHg during sleep are suggestive of nocturnal hypoventilation and should indicate institution of nocturnal NIV [6, 16, 20, 88]. Apart from hypoventilation, the detection of upper airway obstructive events before or after the initiation of NIV might also be of prognostic significance in certain diseases, e.g. ALS [99, 100]. As a rule, initiation of NIV should not be delayed when abnormal findings on oxicapnography combined with morning signs of nocturnal hypoventilation are detected [88].

## 6. Respiratory Management of Patients with Neuromuscular Diseases

The aim of respiratory management of patients with NMDs is threefold: ventilatory support, cough augmentation, and lung volume recruitment. Ventilatory support assists the function of the feeble respiratory muscles and stabilizes gas exchange; cough augmentation improves cough flows and supports secretion clearance; and lung volume recruitment aims to avert functional decline as a result of atelectasis and chest wall contractures [76, 101].

Ventilatory support can be provided either via a noninvasive interface (e.g. nasal, oronasal mask or a mouthpiece) or invasively via a tracheostomy. Although, ventilatory support was initially applied in the form of negative pressure ventilation to treat patients with poliomyelitis in the 1950s, it is now most commonly administered in the form of positive pressure ventilation and is the treatment of choice for patients with NMDs and acute or chronic ventilatory failure [5, 6, 88, 91, 102].

The mechanisms which account for the beneficial effect of NIV on respiratory physiology of patients with NMDs remain unclear. Possible mechanisms include the unloading of the respiratory muscles, the resetting of the respiratory center and improvement in lung mechanics [103]. Nickol et al. suggested that the main benefit of NIV in restrictive lung diseases (kyphoscoliosis and neuromuscular diseases) is associated with the resetting of the chemoreceptors to a lower level of PCO_2_, probably due to reduced renal retention of bicarbonates [104].

Many authorities suggest that NIV should be started in patients with symptoms of nocturnal hypoventilation (e.g. headache, fatigue, daytime sleepiness) plus abnormal findings in oximetry or capnometry (as discussed above). Other indications include a reduced VC (e.g. <50%), decreased respiratory muscle pressures (e.g. SNIP < 40 cm H_2_O or MIP < 65 cm H_2_O) or a daytime PCO_2 _> 45 mmHg [16, 90–92, 105, 106].

It should be noted that tracheostomy ventilation used to be the standard treatment for patients with prolonged or total ventilator dependence and for those with impaired secretion clearance due to weak cough; however the combination of nasal or mouthpiece ventilation (MPV) with cough augmentation strategies has made this option obsolete for the majority of patients with NMDs, except for those with advanced bulbar disease [107–110].

NIV should be administered early during the course of the disease to treat patients with symptomatic sleep hypoventilation. In a randomized controlled trial, which compared NIV to supportive care in normocapnic patients with neuromuscular and chest wall disease and sleep hypoventilation, patients who did not receive NIV experienced faster deterioration, earlier establishment of daytime hypercapnia and more symptoms [3].

Several studies have suggested that NIV can prolong survival in patients with NMDs. A randomized controlled trial, in patients with ALS comparing NIV to supportive care, noted that NIV offered a survival advantage of 205 days, although this benefit was limited to nonbulbar patients. However, some indices of quality of life improved both in bulbar and nonbulbar patients [111]. In addition, a recent retrospective cohort study also observed an overall survival advantage of 15 months for both bulbar and nonbulbar patients with ALS who received treatment with NIV [112]. Simonds et al. conducted an observational study of 23 patients with DMD and diurnal and nocturnal ventilatory failure, who received NIV via nasal mask, and observed 1½ year survival rates of 73% and 85%, respectively [113]. A retrospective study in the total populations of DMD patients in Denmark reported a decrease in mortality from 4.7% to 2.6% per 100 years between 1977 and 2001, which was attributed to an increase in the use of mechanical ventilation from 0.9% to 43.4% during the same period [114].

Institution of NIV in patients with NMDs also improves daytime symptoms, quality of life and parameters of gas exchange. In a prospective observational study of ALS patients treated with NIV for 1 month, nocturnal oxygen and carbon dioxide improved in both bulbar and nonbulbar patients, while sleep quality and electroencephalographic indices of sleep fragmentation improved only in the nonbulbar subgroup [115]. An improvement in sleep and respiratory outcomes which persisted for 15 months was also noted in a retrospective study of bulbar and nonbulbar ALS patients treated with NIV [116]. In the study by Simonds et al., institution of NIV in patients with DMD was associated with an improvement in arterial blood gas values, which persisted throughout the treatment period, while the quality of life was equivalent to other groups of patients with nonprogressive respiratory disorders also treated with NIV [113]. Similarly, in patients with spinal muscular atrophy, NIV normalized sleep architecture and improved respiratory parameters, as well as symptoms of sleep-disordered breathing [117, 118]. In a study of 20 patients with various NMDs, application of NIV was associated with amelioration of the respiratory disturbance index, oxygen saturation, and transcutaneous PCO_2_ as well as indices of daytime sleepiness, as Epworth Sleepiness Scale and Multiple Sleep Latency Test [119].

In the majority of the aforementioned studies [115–119] NIV titration was performed based on sleep oximetric or polysomnographic readings, while relatively low-span bilevel settings (<18 cm H_2_O) were used. However, other authors have criticized this strategy as inadequate to effect complete respiratory muscle rest. These experts prefer the term “noninvasive ventilatory support (NVS)” to emphasize the role of this modality in substituting the respiratory muscles in the work of breathing [5, 6]. Their alternative approach does not employ sleep studies (e.g. polysomnography) for NIV initiation and titration. In fact, it consists of initially implementing NVS during sleep based on reduced supine VC and symptoms of sleep hypoventilation (fatigue, morning headaches, somnolence etc) using either tidal volumes at the range of 0.8–1.5 L in the volume-preset mode or high-span drive pressures (18–25 cm H_2_O) in pressure-preset modes with a back-up respiratory rate commonly set to 12–14 breaths/minute; single limb circuits with true exhalation valves and nasal or oronasal or lipcover interfaces are commonly used. NVS is subsequently applied most of the day during wakefulness when the patient develops symptoms of breathlessness most commonly in volume-preset mode with an angled 15 mm mouthpiece used as interface. This set up allows the patient vary the inhaled tidal volume according to his needs and air-stack ad lib. Nasal masks can be alternatively used for patients with weak mouth muscles who are unable to grab the mouthpiece, while pressure-preset modes can be employed for patients who are unable to air-stack (e.g. infants). This approach is complemented by advanced secretion management strategies i.e., air-stacking and mechanical insufflation exsufflation (MI-E) and also incorporates specifically designed protocols to decannulate tracheostomized patients to 24-hour full ventilatory support [5,6]. According to these protocols, an assisted PECF > 160 L/min effected by a combination of MI-E and abdominal thrust is associated with a very high rate of tracheostomy decannulation success [110, 120].

The aim of the NVS strategy is to avert the use of invasive tubes (tracheostomies), improve survival rates and quality of life and reduce the number of hospital admissions [5, 6, 121]. Thus, in a study reporting on postpolio survivors, MPV was used for 1.028 patient-years (or 14.8 years per patient) for a total mortality of 1 per 60.5 patient-years [122]. In addition, a study on DMD patients under treatment with continuous NVS and mechanically assisted coughing plus cardiac interventions, observed a 50% survival rate of 39.6 months for the continuous NVS group versus 18.1 and 28.9 months for historical patients treated with supplemental oxygen or tracheostomy, respectively [61]. Last, in a study reporting on the use of MPV in 42 final stage DMD patients, 51% of the patients were alive at 7 years post implementation and 50% survived to a mean age of 31 [123]. In addition, the application of continuous NVS plus MI-E to children with SMA 1 was associated with improved survival (sometimes surpassing 20 years of age) and better quality of life for both patients and caregivers [124, 125]. Other authors have also reported on patients with ALS without symptoms of airway spasticity who have been using continuous NVS for many years before tracheostomy [6]. Moreover, in a study by Tzeng and Bach which included neuromuscular patients with PECF of less than 270 L/min or at least 1 episode of respiratory decompensation, the combination of NVS with cough augmentation techniques was associated with a significant reduction in total admissions and the duration in hospital stay due to respiratory exacerbations [68].

For neuromuscular patients with weak cough and reduced cough flows, various cough augmentation strategies should be employed. These techniques could assist either the inspiratory or the expulsive cough phase or both. The former consist of various breath-stacking or lung insufflation maneuvers (see above), while the later consist of manual abdomen or thoracic compression maneuvers [75]. Manually assisted cough with or without breath-stacking or lung insufflation maneuvers should be taught in all patients with PECF < 270/L/min. For patients with PECF < 160/L/min, the use of mechanical insufflation-exsufflation (MI-E) which augments both inspiratory and expulsive cough phases, should be considered [92, 105, 106, 126, 127].

Lung volume recruitment employs the same lung insufflation strategies used for cough augmentation and in fact these two strategies complement each other. Lung volume recruitment is based on achieving maximal lung inflation either with the use of air-stacking techniques or with a MI-E device [76, 128]. Standard lung volume recruitment sessions consist of performing 3–5 (or even more) lung inflation maneuvers twice daily [76, 128]. In cohort of 282 patients with various NMDs, lung volume recruitment applied over several years resulted in increased MIC and LIC, despite a decrease in VC, indicating a higher number of recruitable lung units [76]. In a study including 151 patients with DMD, lung volume recruitment delayed the maximal VC decline by at least 5 years compared to previously reported series [129]. In addition, in a retrospective study of 16 individuals with DMD followed over a median of 6 years, the prescription of lung volume recruitment decreased the rate of VC decline from 4.5% predicted/year to 0.5% predicted/year [128]. McKim et al. also reported retrospectively an 89% decrease in VC decline over a period of 78 months follow-up in 22 DMD patients who were prescribed lung volume recruitment sessions [130]. In another retrospective study on a mixed population of 21 patients with NMDs, the regular use of the MI-E device resulted in improvement in VC by 28% during the first year and stabilization during the second year [131].

In the case of acute respiratory failure (ARF) in patients with NMD, NIV, and aggressive secretion management with MI-E should be introduced. Importantly, NIV should be considered in patients with NMDs who present with an acute illness and exhibit tachypnea (respiratory rate >20/min), even if hypercapnia is absent, especially in those with a known VC < 1 L [132, 133]. Adequate hydration should be ensured and the clinician should have a low threshold for administering antibiotics, particularly when there is high probability of an underlying chest infection [134]. The role of NIV combined with MI-E has been investigated in a prospective non-controlled study involving 16 children with NMDs and acute respiratory failure treated in a pediatric intensive care unit [135]. Hypercapnic respiratory failure was present in 15 cases (mean PaCO_2_ 73.2 mmHg), whereas one had experienced hypoxemic ARF. NIV and MI-E were successfully delivered in 12 cases (75%) with a significant drop in PaCO_2_ levels (>20 mmHg). Interestingly, all patients, including those who were invasively ventilated after intubation, were discharged from the hospital without the need for tracheostomy [135]. Nevertheless, failure of NIV and secretion management strategies (e.g. in the case of severe upper airway obstruction, cranio-facial abnormalities, or coma) should not delay early initiation of invasive ventilation.

## 7. Conclusions

Patients with neuromuscular diseases are at high risk for respiratory complications during the course of their disease. Early recognition of respiratory impairment is vital and a multidisciplinary approach should be adopted in order to provide the best optimal treatment. Noninvasive positive pressure ventilation and advanced cough management strategies are currently the standard of care in neuromuscular diseases as they improve survival, sleep, and quality of life.
